# Supplementary material for: Targeted enhancement of flotillin-dependent endocytosis augments cellular uptake and impact of cytotoxic drugs
Source: Sci Rep. 2019 Nov 28;9:17768. doi: 10.1038/s41598-019-54062-9 (PMC6882852; doi:10.1038/s41598-019-54062-9)
Supplement: Supplementary file 1 — Supplemental Information [file 41598_2019_54062_MOESM1_ESM.pdf]

Targeted enhancement of flotillin-dependent endocytosis augments cellular uptake and impact of cytotoxic drugs

Farnaz Fekri, John Abousawan, Stephen Bautista, Laura Orofiamma, Roya M. Dayam, Costin N. Antonescu, and Raffi Karshafian

Supplemental Information

This Supplemental Information file contains

Table S1
Figure S1
Figure S2
Figure S3
Figure S4
Figure S5
Figure S6
Figure S7
Figure S8

**siRNA name (target)**	**Sense strand sequence**	**Antisense strand sequence**
non-targeting control	CGU ACU GCU UGC GAU ACG GUU	CGT ACT GCT TGC GAT ACG GUU
DHHC5	CUG UGA AGA UCA UGG AUA AUU	UUA UCC AUG AUC UUC ACA GUU
Fyn	AGG AAG AGC UCU GAA AUU AUU	UAA UUU CAG AGC UCU UCC UUU
flotillin1	UGG CCA AGG CAC AGA GAG AUU	UCU CUC UGU GCC UUG GCC AUU
flotillin2	GGA UGA AGC UCA AGG CAG AUU	UCU GCC UUG AGC UUC AUC CUU

Table S1. Sequences of custom siRNA oligonucleotides used in this study. Shown are the sense and antisense sequences for each siRNA oligonucleotide used in this study.

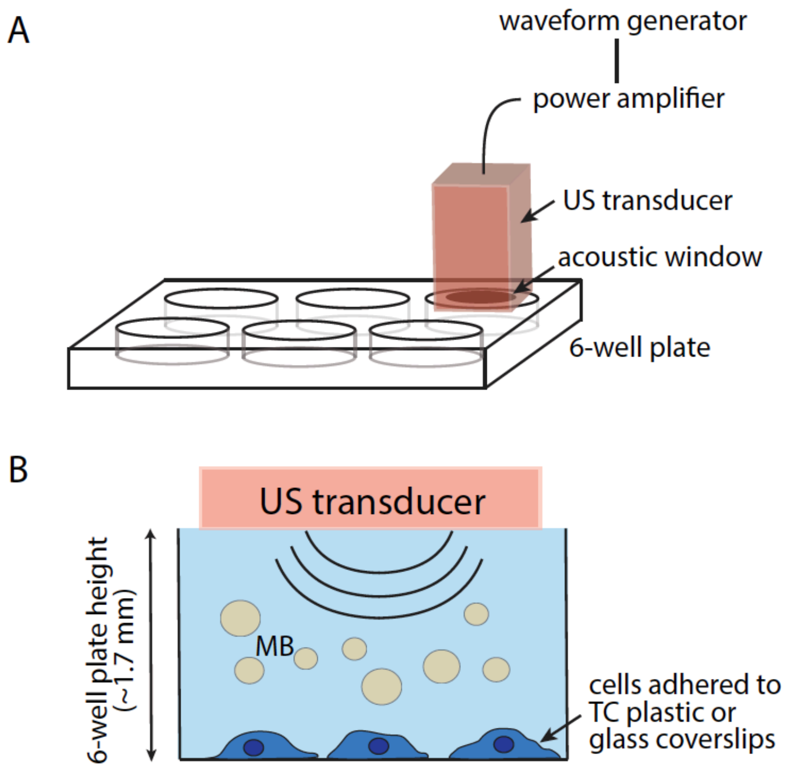

Figure S1. Diagram of the configuration of ultrasound treatment system for adherent cells. (A) Shown is the configuration of the ultrasound waveform generator, power amplifier and ultrasound (US) transducer relative to the 6-well plate on which the cells subjected to treatment are grown. The acoustic window is positioned at the interface of the transducer element and a well of the 6-well plate, the latter which is largely filled (~13mL) with media. (B) Shown is a diagram depicting the side (lateral) view of a well from a 6-well plate subjected to ultrasound and microbubble treatment in this configuration. The ultrasound emitted by the transducer propagates through the aqueous solution (media, shown in blue) containing microbubbles (beige), and adherent cells are grown either on TC plastic or glass coverslips at the bottom of the well.

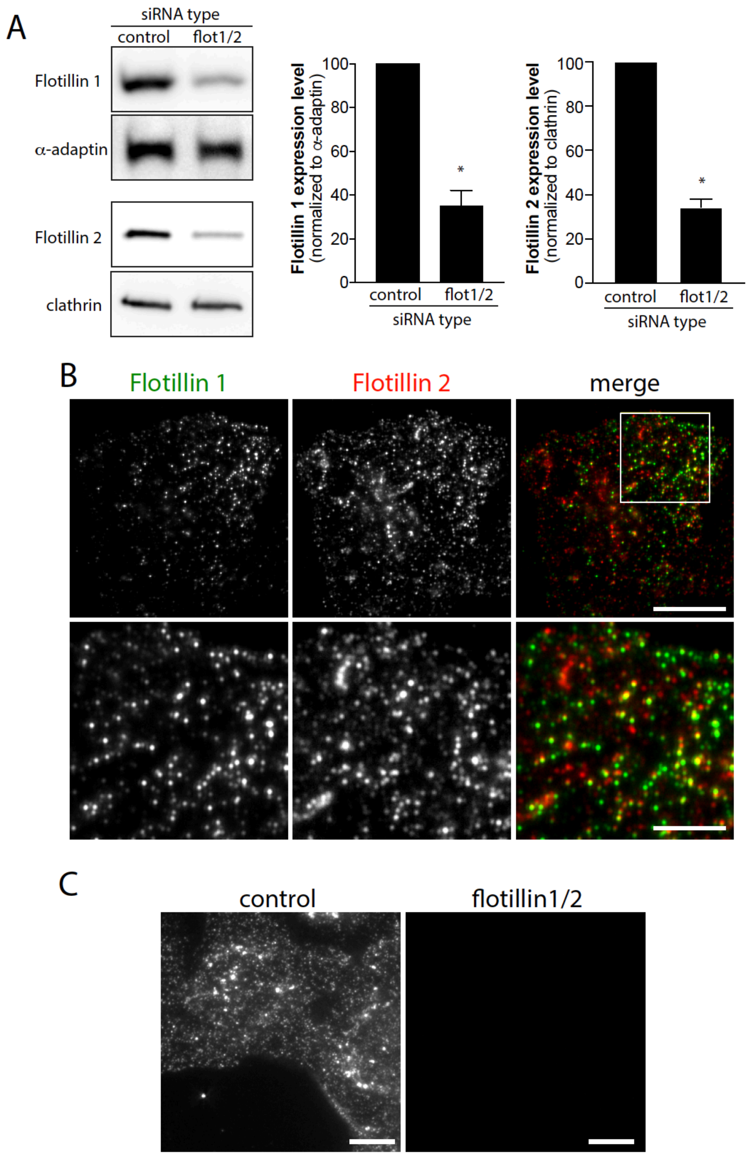

Figure S2. Flotillin knockdown and immunofluorescence detection. (A) RPE cells were transfected with siRNA targeting flotillin-1 and -2 (flotillin) or non-targeting siRNA (control). Whole-cell lysates were resolved by SDS-PAGE and subjected to immunoblotting to detect flotillin-1, flotillin-2, clathrin heavy chain (clathrin, loading control) or alpha-adaptin (AP2, loading control). Shown (left panels) are representative immunoblots and the mean ± SE of flotillin-1 or flotillin-2 detected by this method (right panels). This demonstrates effective knockdown of flotillin1 by siRNA silencing. Full blot images are shown in Figure S8. (B) RPE cells were subjected to immunofluorescence staining to detect flotillin-1 and flotillin-2 and then subjected to imaging by total internal reflection fluorescence microscopy (TIRF-M). Shown are representative fluorescence micrographs that show the extensive co-localization of flotillin-1 and flotillin-2, scale 10 μm. This indicates that the vast majority of flotillin structures in these cells are positive for both flotillin-1 and flotillin-2. (C) RPE cells were transfected with siRNA targeting flotillin-1 and -2 (flotillin) or non-targeting siRNA (control), followed by immunofluorescence staining to detect flotillin-1, then subjected to imaging by TIRF-M. Shown are representative fluorescence micrographs, scale 10 μm, which indicate the specificity of flotillin-1 staining for flotillin-1.

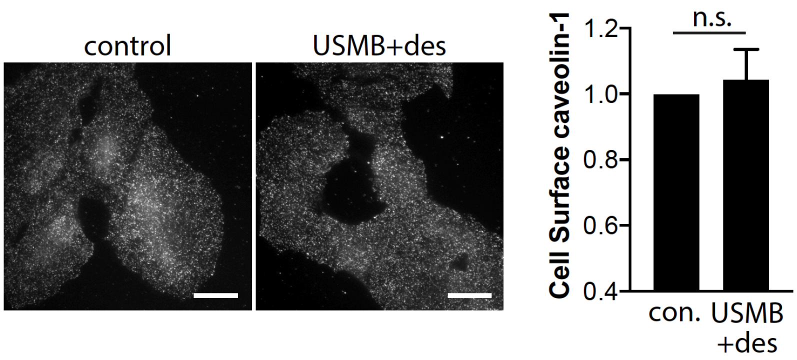

Figure S3. Cell surface caveolin-1 levels are not altered upon treatment with USMB + desipramine. RPE cells were treated with 50 μM desipramine for 60 min followed by USMB treatment, as indicated (identical treatment conditions to experiments used top probe cell surface flotillin levels, e.g. as shown in Fig. 2E, 3A, 5, 6A. Following treatments and a 30 min incubation, cells were fixed and subjected to immunofluorescence staining of caveolin-1, and then imaged by TIRF-M. Shown in are representative TIRF-M fluorescence micrographs, scale 20 μm (left panels). Caveolin-1 intensity in each cell in TIRF-M images was quantified to determine cell surface caveolin-1 levels, and these measurements are shown in the right panel as mean ± SE. n = 3 independent experiments. n.s., not significantly different.

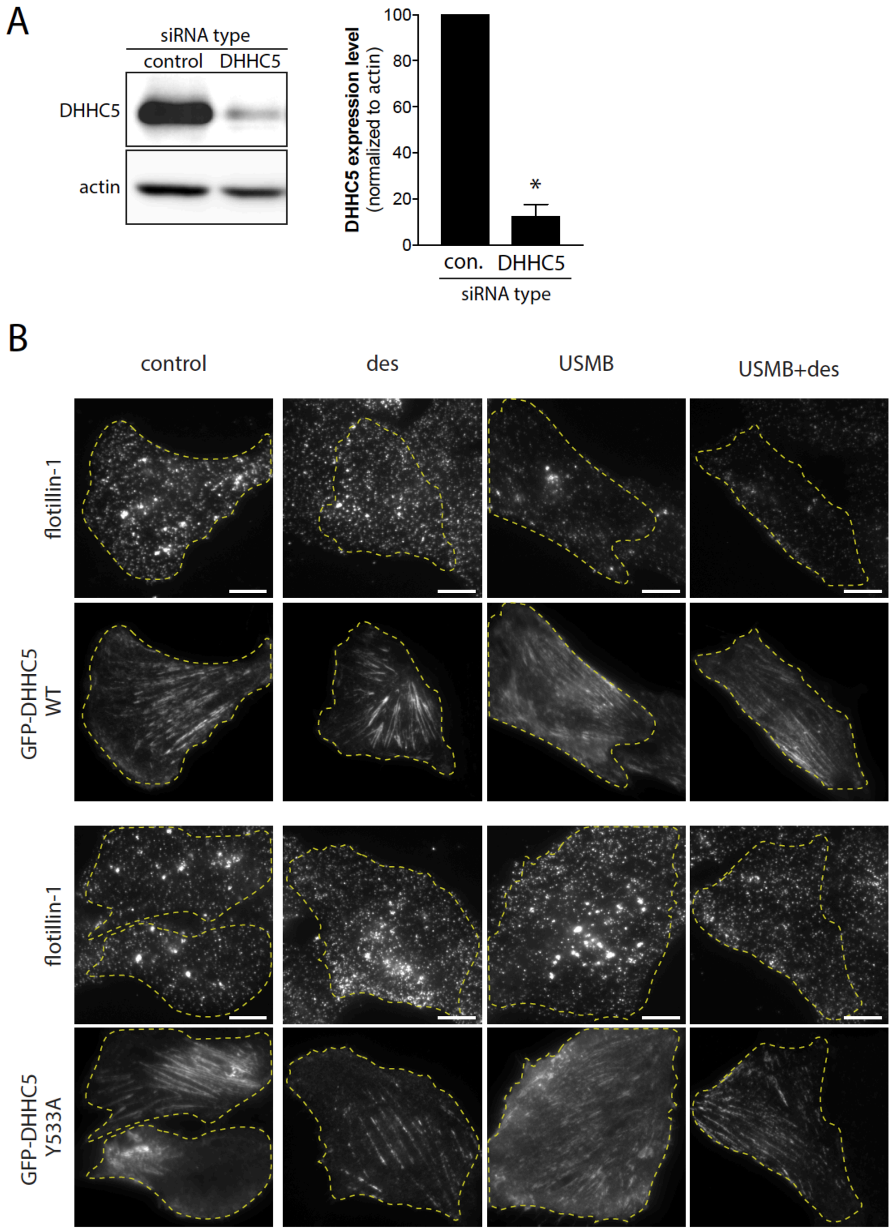

Figure S4. DHHC5 knockdown and transfection. (A) RPE cells were transfected with siRNA targeting DHHC5 or non-targeting siRNA (control). Whole-cell lysates were resolved by SDS-PAGE and subjected to immunoblotting to detect DHHC5 or actin (loading control). Shown (left panels) are representative immunoblots and the mean ± SE of DHHC5 detected by this method (right panels). This demonstrates effective knockdown of DHHC5 by siRNA silencing. Full blot images are shown in Figure S8. (B) RPE cells were transfected with cDNA encoding either wild-type (WT) or Y533A mutant DHHC5, fused to eGFP. Following transfection, some cells were treated with 50 μM desipramine for 60 min followed by USMB treatment, as indicated. Following a subsequent 30 min incubation, cells were then fixed and subjected to immunofluorescence staining of flotillin-1, and then imaging by TIRF-M. Shown are representative TIRF-M fluorescence micrographs, scale 10 μm. Transfected cells are outlined, based on eGFP signal. These images show the eGFP-DHHC5 expression of cells shown in **Figure 5**.

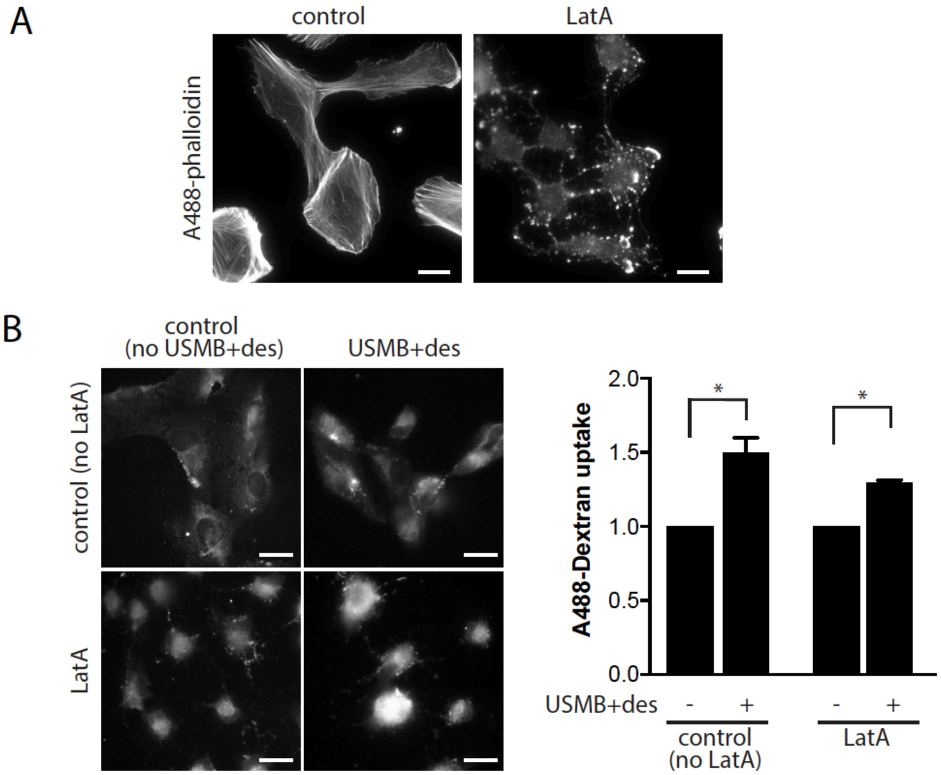

Figure S5. The increase in fluid-phase internalization triggered by USMB+desipramine treatment is largely unaffected by latrunculin treatment. RPE cells were treated with 0.5 μM Latrunculin A (LatA) or vehicle control (DMSO, 0.1% vol/vol) for 60 min as indicated. (A) Following LatA treatment, cells were fixed and subjected to staining with Alexa488(A488)-phalloidin. Shown are single z-plane fluorescence micrographs obtained by spinning-disc confocal microscopy of A488-phalloidin fluorescence. This demonstrates that the LatA treatment conditions are effective to achieve robust disruption of the actin cytoskeleton. (B) Following LatA treatment, some cells were treated with 50 μM desipramine for 60 min followed by USMB treatment, as indicated. Following these treatments, all cell samples were incubated with 10 μg/mL A488-dextran for 30 min, fixed, and imaged by widefield epifluorescence microscopy. Shown in are representative fluorescence micrographs showing A488-dextran in cells of each treatment (left panel), scale 20 μm. Also shown (right panel) are the mean ± SE of total cellular A488-dextran in teach condition n = 3 independent experiments. *, $p < 0.05$. As LatA treated cells exhibited an altered morphology and occupied a reduced surface area, direct comparison of the mean fluorescence intensity of control (no LatA) with LatA-treated cells is challenging. As such, results from control (No LatA) and LatA treated cells are each normalized to their corresponding condition in which cells were not subjected to USMB+des.

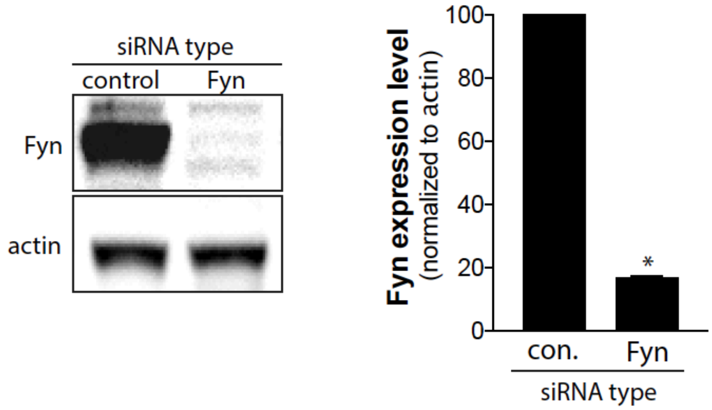

Figure S6. Fyn knockdown. RPE cells were transfected with siRNA targeting Fyn or non-targeting siRNA (control). Whole-cell lysates were resolved by SDS-PAGE and subjected by immunoblotting to detect Fyn or actin (loading control). Shown (left panels) are representative immunoblots and the mean ± SE of Fyn detected by this method (right panels). This demonstrates effective knockdown of Fyn by siRNA silencing. Full blot images are shown in Figure S8.

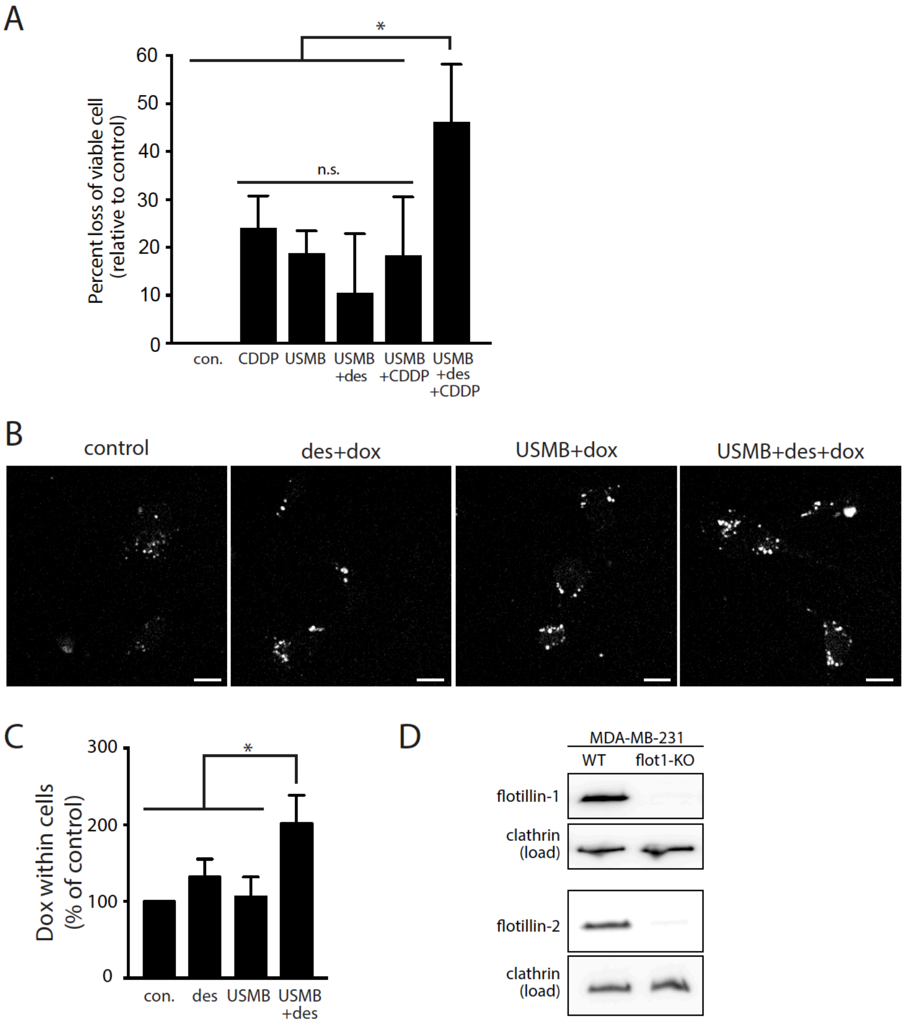

Figure S7. Flotillin knockout cells and contribution of flotillin to cell viability in USMB-treated cells. (A) RPE cells were treated with 50 μM desipramine for 60 min followed by USMB treatment, as indicated. Following treatments, some cells were incubated with 30 μM cisplatin (CDDP) for 2 h, as indicated, followed by washing and incubation in growth media (no drugs). 24 h after USMB and/or cisplatin exposure, cell viability was assessed by crystal violet assay, and shown are the mean ± SE of the percent reduction in the total viable cells, relative to that of control (not treated with CDDP, USMB or desipramine). n = 3 independent experiments. *, $p < 0.05$. (B-C) RPE cells were treated with 50 μM desipramine for 60 min followed by USMB treatment, as indicated. Following treatments, cells were incubated with 30 μM doxorubicin for 2 h (as shown), followed by washing and incubation in growth media (no drugs) for 24 h, after which cells were subjected to widefield epifluorescence microscopy to detect doxorubicin fluorescence within cells. Shown in (B) are representative fluorescence micrographs of doxorubicin fluorescence, and in (C) the mean ± SE of cellular doxorubicin fluorescence. n = 3 independent experiments. *, $p < 0.05$. This indicates that cells treated with USMB+desipramine retain more chemotherapeutic drugs 24 h after exposure to these drugs compared to control cells (cells not treated with USMB+desipramine). (D) Whole cell lysates from MDA-MB-231 wild-type (WT) cells or MDA-MB-231 cells subjected to CRISR/Cas9 genome editing (to knock out flotillin-1) were resolved by SDS-PAGE and subjected by immunoblotting to detect flotillin-1, flotillin-2 or clathrin heavy chain (loading control). Shown are representative immunoblots demonstrates effective knockout of flotillin in MDA-MB-231-flot-KO cells. Full blot images are shown in Figure S8.

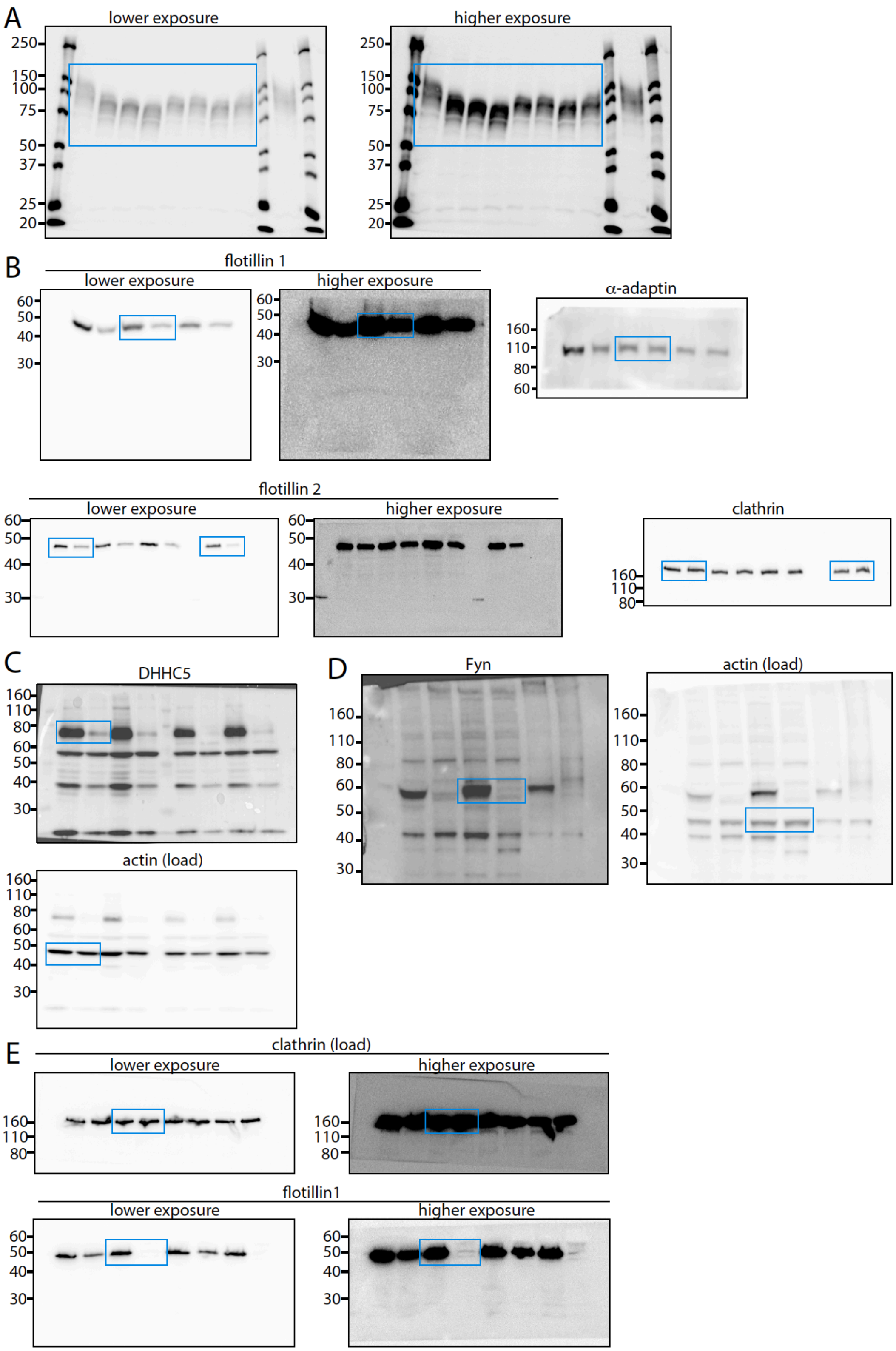

Figure S8. Full-panel images of immunoblots. Shown are the full images for the immunoblots shown in this study, as follows, by panel: (A) Full images for western blot shown in Figure 4A. (B)

Full images for western blot images shown in Figure S2A (flotillin-1 and -2, and corresponding loading controls for α-adaptin and clathrin heavy chain (clathrin), as well as for Figure S8D (flotillin-2 and clathrin). (C) Full images for western blot images shown in Figure S4A. (D) Full images for western blot images shown in Figure S6. (E) Full images for western blot images shown in Figure S7D. Blue boxes represent cropped portions of each image shown in indicated figures. Shown for each are the Mr values for the molecular weight markers, which were Precision Plus Protein™ All Blue Prestained Protein Standards (Bio-Rad) for immunoblots shown in A and Novex Sharp Pre-stained Protein Standard (Thermo Fisher Scientific) shown in B-E. Some full-panel immunoblot membranes (panels B and E) were cut between the 60 and 80 Mr markers prior to antibody labeling. Probing for actin (loading control) was performed subsequent to initial blotting for DHHC5 or Fyn, on the same membrane in panels C and D, respectively.
